# Effect of Physical Therapy on Inflammatory Biomarkers in Knee Osteoarthritis: A Systematic Review and Meta-Analysis of Randomized Controlled Trials

**DOI:** 10.3390/jcm15135288

**Published:** 2026-07-07

**Authors:** Abdulraouf H. Ayoub, Ahmed A. Alsirhani, Ali M. Alshami

**Affiliations:** 1 Department of Physical Therapy, College of Applied Medical Sciences, Imam Abdulrahman Bin Faisal University, Dammam 31441, Saudi Arabia; alshami@iau.edu.sa; 2 Department of Physical Therapy, Aseer Health Cluster, Ministry of Health, Abha 62523, Saudi Arabia

**Keywords:** osteoarthritis, knee, physical therapy modalities, biomarkers, C-reactive protein, erythrocyte sedimentation rate

## Abstract

**Background/Objectives**: Knee osteoarthritis (OA) is increasingly recognized as a condition with an inflammatory component. Elevated C-reactive protein (CRP) and erythrocyte sedimentation rate (ESR) are associated with disease severity. However, the effects of physical therapy on inflammatory biomarkers remain unclear. This review aimed to evaluate the effects of physical therapy interventions on CRP and ESR in adults with knee OA. **Methods**: Following PRISMA 2020 guidelines, PROSPERO (CRD42024590843), PubMed, MEDLINE, Embase, Scopus, Web of Science, and Google Scholar were searched from inception to 3 November 2024. Randomized controlled trials comparing any physical therapy intervention with a control condition were included. Risk of bias was assessed using the Physiotherapy Evidence Database (PEDro) scale, and certainty of evidence was evaluated using the GRADE approach. Random-effects meta-analyses were performed using standardized mean differences (SMDs) with 95% confidence intervals (CIs). **Results**: Seven RCTs (*n* = 523 participants) were included. Pooled analysis showed a statistically significant reduction in CRP (SMD = −1.05; 95% CI: −1.80 to −0.31; *p* = 0.006); however, substantial heterogeneity was observed (I^2^ = 93%), limiting confidence in the pooled estimate. Certainty of evidence for CRP was rated as moderate. No significant effect was observed for ESR (SMD = −0.20; 95% CI: −0.84 to 0.45; *p* = 0.56; I^2^ = 63%), with low certainty. **Conclusions**: Physical therapy may reduce CRP levels in knee OA; however, substantial heterogeneity limits confidence in the precision and generalizability of this effect. These findings should be interpreted cautiously and considered exploratory. Further well-designed RCTs are required.

## 1. Introduction

Knee osteoarthritis (OA) affects an estimated 22–30% of the global population aged 45 years and older [1]. The prevalence of knee OA is expected to rise significantly in the coming decades, driven by the aging global population and increasing rates of obesity [2]. Individuals with knee OA frequently experience pain, reduced mobility, periods of physical inactivity, and limitations in performing daily activities, all of which contribute to a diminished quality of life [3,4].

Recently, knee OA has been recognized as a complex disease characterized by low-grade inflammation that plays an important role in its pathogenesis [5]. This inflammatory process is characterized by elevated levels of systemic inflammatory markers, such as C-reactive protein (CRP) and erythrocyte sedimentation rate (ESR) [6]. Elevated levels of CRP and ESR have been linked to pain severity, joint stiffness, and radiographic progression of knee OA, suggesting their potential utility as diagnostic and prognostic indicators [7].

The impact of physical therapy on inflammatory biomarkers, specifically CRP and ESR, in individuals with knee OA remains unclear due to limited and heterogeneous evidence. While some studies have reported the potential benefits of certain physical therapy modalities, such as manual therapy, in modulating inflammatory responses reflected in CRP and ESR levels [8], the overall evidence regarding the effectiveness of various physical therapy interventions remains insufficient.

Several systematic reviews have examined the effects of exercise therapy on inflammatory biomarkers in knee osteoarthritis (OA). Bricca et al. (2019) reported nonsignificant pooled effects on CRP [9], while Puts et al. (2023) and Mauri et al. (2025) identified heterogeneous findings with limited quantitative synthesis across physiotherapy modalities [10,11]. However, these reviews primarily focused on exercise, with limited quantitative evaluation of other physiotherapy modalities.

In addition, Anand et al. (2025) synthesized evidence on inflammatory biomarkers in physiotherapy interventions for knee OA but presented findings narratively without quantitative meta-analysis [12]. Furthermore, ESR has not been consistently evaluated in prior meta-analyses despite its routine clinical use.

Therefore, a systematic review and meta-analysis restricted to RCTs and encompassing multiple physiotherapy modalities, with quantitative pooling of both CRP and ESR, remains warranted. Accordingly, the aim of this systematic review and meta-analysis was to quantitatively synthesize evidence from RCTs to determine the effects of physical therapy interventions, compared with control or usual care, on inflammatory biomarkers in individuals with knee osteoarthritis. Specifically, this review focused on CRP and ESR.

## 2. Materials and Methods

### 2.1. Protocol Registration

This systematic review with meta-analysis was conducted and reported according to the Preferred Reporting Items for Systematic Reviews and Meta-Analyses (PRISMA) 2020 guidelines [13] and registered in the International Prospective Register of Systematic Reviews (PROSPERO) (CRD42024590843; on 15 November 2024).

### 2.2. Eligibility Criteria

Eligibility criteria were defined using the Population, Intervention, Comparator, Outcomes, and Study design (PICOS) framework. Studies were included if they enrolled adults (≥18 years) with a clinical and/or radiographic diagnosis of knee OA, as defined by the study authors (e.g., physician diagnosis, American College of Rheumatology criteria, or radiographic evidence such as Kellgren–Lawrence grading). Eligible interventions included any form of physical therapy, such as exercise therapy, manual therapy, electrotherapy, and thermotherapy. The comparator group was any control condition, including usual care or sham intervention. The outcomes of interest were CRP and/or ESR levels measured at baseline and post-intervention. Only published RCTs were included, as they represent a high level of evidence in the hierarchy of medical research and reduce the risk of bias compared with observational designs [14].

Studies were excluded if they were non-English, review articles, conference abstracts, editorials, animal or in vitro studies, or duplicate publications. In cases of duplicates, the most complete or recent version was included.

### 2.3. Search Strategy

A literature search was conducted on 3 November 2024, from inception in the following electronic databases: PubMed, MEDLINE, Embase, Scopus, Web of Science, and Google Scholar. The search was subsequently updated on 21 June 2026, to ensure inclusion of the most recent evidence. The search strategy and keywords were developed by the primary reviewer (A.H.A.) in consultation with a medical librarian, combining both free-text terms and database-specific subject headings (e.g., Medical Subject Headings [MeSH]). The initial strategy was created for PubMed and adapted for each database to account for differences in indexing and controlled vocabulary.

The search strategy included terms related to knee OA (“knee osteoarthritis,” “osteoarthritis of the knee”), physical therapy (“physical therapy,” “physiotherapy,” “exercise therapy,” “manual therapy,” “electrotherapy,” and “rehabilitation”), and inflammatory biomarkers (“C-reactive protein,” “CRP,” “erythrocyte sedimentation rate,” and “ESR”). Synonymous terms were combined using the Boolean operator OR in the electronic search strategy. The search was limited to studies published in English. This restriction was applied due to resource constraints. The detailed search strategy is provided in the Supplementary Materials.

In addition to electronic database searches, the reference lists of all included studies and relevant systematic reviews were manually screened to identify additional eligible studies not captured in the initial search. The database search was conducted independently by two reviewers (A.H.A. and A.A.A.).

### 2.4. Study Selection

Records identified through database searching were imported into Rayyan (Qatar Computing Research Institute) for duplicate removal and screening. Two reviewers independently screened titles and abstracts to identify potentially eligible studies. Full-text articles of relevant records were then assessed independently for eligibility according to the predefined inclusion and exclusion criteria. The study selection process was documented using a PRISMA 2020 flow diagram.

### 2.5. Data Extraction

Data were extracted independently by two reviewers using a standardized data extraction form. Extracted information included study characteristics (authors, year of publication), participant characteristics (sample size, mean age), intervention details (type and duration of treatment), follow-up duration, outcome measures, time points of assessment, and numerical data required for meta-analysis (means, standard deviations, and sample sizes). When necessary, corresponding authors were contacted for missing data.

### 2.6. Risk of Bias Assessment

The methodological quality and risk of bias of the included studies were assessed independently by the two reviewers using the PEDro scale [15]. The PEDro scale assesses internal validity and statistical reporting across 10 items, with total scores ranging from 0 to 10. Studies were ranked according to their scores as follows: good (≥6), fair (4–5), and poor (≤3) [16]. The PEDro scale was selected due to its established validity and widespread use in physiotherapy randomized controlled trials.

### 2.7. Certainty of Evidence Assessment

The certainty of evidence for each meta-analytic outcome was assessed independently by two reviewers using the Grading of Recommendations Assessment, Development and Evaluation (GRADE) approach [17]. The assessment was performed at the outcome level across five domains: risk of bias, inconsistency, indirectness, imprecision, and publication bias. Randomized controlled trials were initially considered high-certainty evidence and were downgraded when concerns were identified in any of the GRADE domains. The certainty of evidence was rated as high, moderate, low, or very low. Summary of Findings tables were generated using GRADEpro Guideline Development Tool (GDT) online version (McMaster University and Evidence Prime). 

Any disagreements between the two reviewers regarding study selection, data extraction, risk of bias assessment, or certainty of evidence assessment were resolved through discussion and consensus. If agreement was not reached, a third reviewer (A.M.A.) was consulted.

### 2.8. Data Synthesis and Analysis

Meta-analyses were conducted when studies reported comparable outcomes and demonstrated sufficient clinical and methodological homogeneity to support quantitative synthesis. A random-effects model was used to pool data due to anticipated heterogeneity between studies. Standardized mean differences (SMDs) with 95% confidence intervals (CI) were calculated for continuous outcomes (CRP and ESR levels) using post-intervention mean values and standard deviations. Post-intervention values were used to ensure consistency across studies reporting outcomes at similar time points.

Heterogeneity was assessed using the I^2^ statistic. I^2^ values of 25%, 50%, and 75% were interpreted as low, moderate, and high heterogeneity, respectively [18]. A subgroup analysis based on the number of sessions per treatment day was conducted to explore potential sources of heterogeneity and was considered exploratory.

Sensitivity analyses were performed by sequentially excluding trials delivering multiple sessions per treatment day to assess the robustness of the pooled estimates. All analyses were conducted using Review Manager (RevMan) software version 5.4 (Cochrane Collaboration, Oxford, UK).

## 3. Results

### 3.1. Study Selection

**Figure 1:**
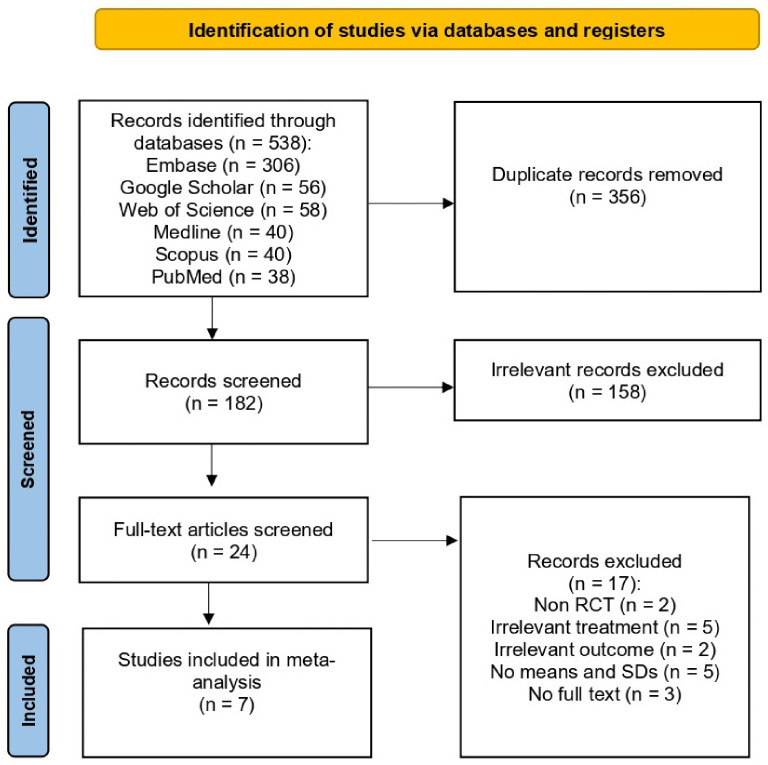
PRISMA flow diagram of the selection and screening of RCTs.

Figure 1 presents the PRISMA flow diagram illustrating the study selection process. A total of 541 records were identified through six databases, including three additional records identified in the updated search conducted on 21 June 2026. After removing 356 duplicate records, 185 records remained and were screened by title and abstract. Of these, 25 full-text articles were assessed for eligibility. Following the application of the inclusion and exclusion criteria, seven randomized controlled trials (RCTs) were included in the meta-analysis.

### 3.2. Description of Included Studies

Table 1 provides an overview of the characteristics of the included RCTs. Published between 2013 and 2024, these studies collectively analyzed 523 participants, with a mean age of 63.1 years (range: 41–69). Four RCTs reported gender distribution, with 165 (43.7%) males and 213 (56.3%) females, whereas three RCTs did not report gender details [19,20,21].

The interventions included various physical therapy treatments, such as whole-body electromyostimulation (WB-EMS) [19], acupuncture with standard care [22], home-based remote rehabilitation focusing on lower extremity strengthening [20], auricular bean pressing added to routine rehabilitation [23], Maitland’s mobilization techniques [8], isokinetic and aerobic exercise regimens [21], and exercises combined with diclofenac sodium [24].

**Table 1:** Characteristics of the included randomized controlled trials.

Author (Year)	Sample Size (Total)	Age (Mean ± SD)	Treatment	Intervention Frequency and Duration	Follow-Up
Kelmendi et al. (2024) [19]	EG: 36 CG: 36 (72)	EG: 58.3 ± 7.2 CG: 57.9 ± 7	EG: 20 min/week WB-EMS CG: 6 PT sessions/week	1×/week for 29 weeks	Week 29
Lee et al. (2023) [20]	EG: 15 CG: 16 (31)	EG: 65.6 ± 3.7 CG: 68.3 ± 4.8	EG: Home-based strengthening CG: No intervention	3×/week for 8 weeks	Week 8
Lin et al. (2023) [23]	EG: 50 CG: 50 (100)	EG: 69.3 ± 5.1 CG: 67.2 ± 5.3	EG: Routine rehab + auricular bean pressing CG: Routine rehab	3×/day for 4 weeks	Week 4
Samut et al. (2015) [21]	EG I: 15 EG II: 14 CG: 13 (42)	EG I: 63.2 ± 7.1 EG II: 61.6 ± 5.6 CG: 62.7 ± 6.4	EG I: Isokinetic EG II: Aerobic CG: No exercise	3×/week for 6 weeks	Week 6
Shabbir et al. (2022) [8]	EG: 35 CG: 35 (70)	EG: 59.9 ± 3.0 CG: 61.3 ± 5.3	EG: Maitland mobilization CG: Heat, exercises, TENS	3×/week for 12 weeks	Week 18
Wei et al. (2024) [22]	EG: 54 CG: 54 (108)	EG: 61.1 ± 7.1 CG: 61.1 ± 6.6	EG: Acupuncture + standard care CG: Sham acupuncture + standard care	6×/week for 4 weeks	Week 4
Zhang et al. (2013) [24]	EG: 50 CG: 50 (100)	EG: 53 ± 5.6 CG: 52.3 ± 7	EG: DS + exercise CG: DS only	2×/day, 4 days/week for 4 weeks	Week 4

Abbreviations: CG, comparison group; DS, diclofenac sodium; EG, experimental group; min, minutes; PT, physical therapy; WB-EMS, whole-body electromyostimulation.

Comparison groups received interventions such as standard physical therapy [8,19,23], sham acupuncture [22], no intervention [20,21], or diclofenac sodium without exercise [24]. Intervention durations ranged from 4 to 29 weeks, with treatment frequencies varying from daily to once weekly.

All RCTs assessed CRP as an inflammatory biomarker, and two RCTs assessed ESR [20,22]. Additional clinical outcome measures included pain intensity using the visual analog scale (VAS) and the numeric pain rating scale (NPRS); functional capacity assessed by the Western Ontario and McMaster Universities Osteoarthritis index (WOMAC), the Lysholm Knee Scoring Scale (LKSS), the Lequesne Index, the Five-Times Sit-to-Stand Test (FTSST), the Timed Up and Go (TUG) test, the 30-Second Sit-to-Stand (30 s STS) test, and the 6-Minute Walk Test (6 MWT); and/or knee range of motion (ROM) and strength. Detailed information on the assessed biomarkers, additional clinical outcomes, and the main findings of the included studies are presented in Table 2.

### 3.3. Risk of Bias

The methodological quality assessment is presented in Table 3. PEDro scores ranged from 5 to 8 (mean ± SD of 6.7 ± 1.4). Five RCTs were categorized as good methodological quality [8,19,20,22,23], and two were categorized as fair [21,24]. Blinding of participants and therapists was absent in most studies. The two trials rated as fair did not implement concealed allocation, blinding of participants and therapists, or intention-to-treat analysis.

### 3.4. Certainty of Evidence

The GRADE assessment indicated that the certainty of evidence was moderate for CRP due to substantial heterogeneity. The certainty of evidence for ESR was rated as low because of inconsistency and imprecision. The Summary of Findings table is presented in Table 4.

### 3.5. Results of Synthesis

**Figure 2:**
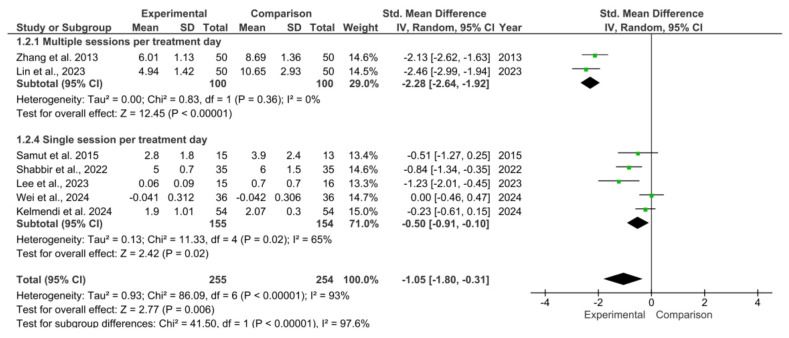
Forest plot of the effect of physical therapy on CRP levels including Zhang et al. 2013 [24], Lin et al. 2023 [23], Samut et al. 2015 [21], Shabbir et al. 2022 [8], Lee et al. 2023 [20], Wei et al. 2024 [22], and Kelmendi et al. 2024 [19].

All seven RCTs (*n* = 523 participants) evaluated the effect of physical therapy on CRP levels. Physical therapy was associated with a reduction in CRP levels (SMD = −1.05; 95% CI: −1.80 to −0.31; *p* = 0.006). However, very high heterogeneity was observed across studies (I^2^ = 93%) (Figure 2). The wide CI and substantial heterogeneity indicate considerable uncertainty around the magnitude of the pooled effect. According to conventional interpretation of standardized mean differences (0.2 small, 0.5 moderate, 0.8 large) [25,26], the pooled effect size suggests a large reduction in CRP levels; however, this interpretation should be considered cautiously due to the observed heterogeneity.

Subgroup analysis based on the number of sessions delivered per treatment day suggested variation in effect estimates. The multiple sessions per treatment day subgroup (two RCTs; *n* = 200) showed a larger effect size for CRP reduction (SMD = −2.28; 95% CI: −2.64 to −1.92; *p* < 0.00001) with no observed heterogeneity (I^2^ = 0%). In contrast, the single-session per treatment day subgroup (five RCTs; *n* = 309) demonstrated a smaller reduction in CRP (SMD = −0.50; 95% CI: −0.91 to −0.10; *p* = 0.02), with moderate heterogeneity (I^2^ = 65%).

Sensitivity analyses were conducted by sequentially excluding the trials delivering multiple sessions per treatment day. Exclusion of either trial individually did not substantially change the direction or magnitude of the pooled effect estimate. The pooled effect remained statistically significant in both cases (*p* = 0.02), and heterogeneity remained high (I^2^ = 90–92%). When both trials were excluded, the pooled effect size was attenuated (SMD = −0.46; 95% CI: −0.85 to −0.07; *p* = 0.02). Heterogeneity decreased from 93% to 65%, suggesting that the trials delivering multiple sessions per treatment day contributed to both the magnitude of the pooled effect and the observed heterogeneity.

**Figure 3:**
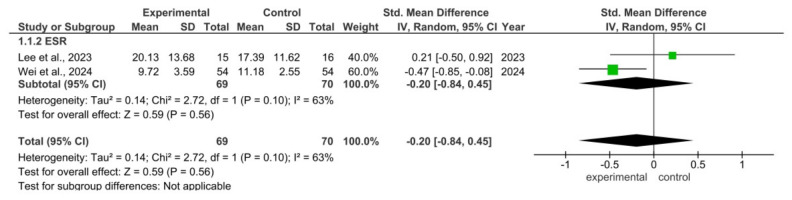
Forest plot of the effect of physical therapy on ESR including Lee et al. 2023 [20], Wei et al. 2024 [22].

For ESR, two RCTs, including 139 participants, were analyzed. The pooled analysis did not demonstrate a statistically significant effect on ESR following physical therapy (SMD = −0.20; 95% CI: −0.84 to 0.45; *p* = 0.56), and moderate heterogeneity was observed (I^2^ = 63%) (Figure 3).

## 4. Discussion

This systematic review and meta-analysis synthesized evidence from RCTs to evaluate the effects of physical therapy on inflammatory biomarkers, namely CRP and ESR, in individuals with knee OA. The pooled analysis suggested that physical therapy was associated with a reduction in CRP levels compared with control interventions. However, this finding was accompanied by very high heterogeneity (I^2^ = 93%) and a wide CI, indicating substantial uncertainty regarding the magnitude and consistency of the observed effect. In contrast, no clear effect was observed for ESR based on the limited number of available trials.

Although the pooled standardized mean difference for CRP was large according to conventional interpretation thresholds [25,26], this magnitude should be interpreted cautiously. The very high heterogeneity suggests that between-study variability was unlikely to be attributable to chance alone and instead reflects considerable clinical and methodological diversity across trials. Differences in intervention type (exercise, manual therapy, electrotherapy, acupuncture), treatment frequency, overall program duration (4–29 weeks), participant characteristics, and timing of biomarker assessment likely contributed to the observed inconsistency. Accordingly, while a reduction in CRP was observed, the certainty regarding the precise magnitude of effect remains limited. Although the overall risk of bias was judged as not serious according to the GRADE framework, several included trials did not implement double-blinding or fully report allocation concealment and intention-to-treat analysis. These methodological limitations may have introduced performance or detection bias and could have contributed to variability in effect size estimates across studies. Therefore, the pooled findings should be interpreted in light of both clinical and methodological diversity.

The subgroup analysis indicated that trials delivering multiple sessions per treatment day demonstrated larger reductions in CRP with no observed heterogeneity, whereas single-session protocols showed smaller effects and moderate heterogeneity. However, these findings should be considered exploratory. The small number of studies per subgroup and the absence of formal meta-regression limit the ability to determine whether treatment frequency independently explains the observed differences. Sensitivity analyses further demonstrated that exclusion of higher-frequency trials attenuated the pooled effect size and reduced heterogeneity, suggesting that these trials substantially influenced both the magnitude and variability of the overall estimate. Taken together, the direction of effect for CRP was relatively consistent, but the magnitude varied considerably, limiting precision in estimating the true treatment effect.

Our findings build upon the review by Bricca et al., which evaluated exercise therapy alone and reported nonsignificant reductions in CRP with low certainty of evidence. In contrast, our analysis showed a significant reduction in CRP levels. This discrepancy may relate to the broader range of physical therapy modalities included in the present review. Differences in inclusion criteria and intervention protocols may also explain the inconsistency. However, similar to the previous review, substantial heterogeneity remains a key limitation when interpreting these results.

The physical therapy interventions identified in our review included exercise, joint manual therapy, electrical stimulation, and acupuncture. Several mechanisms may contribute to the observed reduction in CRP in patients with knee OA. For example, exercise exerts systemic anti-inflammatory effects through improved insulin sensitivity [27], promotion of weight management [28], and modulation of cytokine production [29]. Manual therapy and specific exercises can improve joint mechanics, reducing stress on articular tissues and relieving inflammation [30], which may contribute to improved local circulation and modulation of inflammatory mediators [31]. Similarly, acupuncture may further contribute by modulating pro-inflammatory cytokines, including interleukin-1 beta (IL-1β) and tumor necrosis factor-alpha (TNF-α) [32] and inhibiting the p38 mitogen-activated protein kinase signaling pathway [22]. In addition, other physical therapy modalities have been reported to influence inflammatory biomarkers in musculoskeletal conditions [33,34].

The predefined subgroup analysis suggested that interventions delivered more frequently per treatment day were associated with larger reductions in CRP levels. However, this finding should be interpreted with caution. Sensitivity analyses demonstrated that although the overall effect remained statistically significant after excluding the higher-frequency trials, the magnitude of the pooled effect was attenuated and heterogeneity was substantially reduced. This suggests that the number of sessions delivered per treatment day may have contributed to between-study heterogeneity, although the limited number of trials precludes definitive conclusions.

In addition to treatment frequency, substantial variation in overall program duration (ranging from 4 to 29 weeks) may also have contributed to the observed heterogeneity. Longer intervention periods may allow cumulative physiological adaptations, whereas shorter programs may primarily capture acute inflammatory responses. Given the limited number of included trials, a formal meta-regression to examine the independent effects of intervention frequency and duration was not feasible. Therefore, the relative contribution of these factors to the pooled estimates could not be formally determined. To the best of our knowledge, no previous meta-analyses have examined the effects of different physical therapy dosing parameters on CRP or ESR in patients with knee OA.

Our meta-analysis also found that physical therapy interventions did not significantly change ESR levels in patients with knee OA. However, this finding was based on the two RCTs and should therefore be interpreted cautiously. The limited sample size substantially reduces statistical power and increases the likelihood of type II error, meaning that a potentially meaningful effect may not have been detected. The absence of a clear effect may reflect limited statistical power rather than true lack of responsiveness. Additionally, ESR has a broader inflammatory scope compared with CRP [35], and may be less sensitive to localized or short-term physiological changes induced by physical therapy [36]. ESR is also influenced by factors unrelated to joint inflammation, including age, sex, hematologic variables, and comorbid conditions [37], which may further obscure intervention effects.

CRP and ESR are both elevated in knee OA and have been associated with symptom severity and disease progression [5,38]. However, they differ in kinetics and clinical applications. CRP rises within hours of inflammatory stimuli and declines rapidly, making it sensitive to acute changes in OA-related inflammation [9]. In contrast, ESR reflects chronic inflammation, is influenced by plasma protein levels [39], rises more slowly, and remains elevated longer. While CRP is more specific to OA pathophysiology and is linked to radiographic severity and pain progression [40], ESR is broader and less specific and may be affected by anemia, age, or comorbidities [36]. Clinically, CRP’s rapid responsiveness makes it valuable for monitoring treatment efficacy [9], whereas ESR’s low cost and accessibility maintain its utility in resource-limited settings [41]. However, in the absence of validated minimal clinically important difference thresholds for CRP in knee OA, biomarker reductions should not be interpreted as evidence of clinically meaningful improvement. Therefore, changes in CRP should be considered exploratory and not a surrogate for patient-important outcomes such as pain or functional status.

There is growing recognition of inflammation as a key driver in OA pathogenesis, not merely as a consequence of cartilage breakdown [42]. OA-related inflammation is a multifaceted process involving mediators. Our findings suggest that physical therapy may influence systemic inflammatory activity in knee OA. Whether such changes translate into clinically meaningful improvements requires further investigation.

### 4.1. Study Strengths

This review has several strengths, including a comprehensive and systematic search across multiple databases and the restriction to RCTs, which enhances internal validity. Quantitative synthesis through meta-analysis provided pooled effect estimates, and the use of random-effects models accounted for anticipated clinical and methodological heterogeneity across trials. Furthermore, application of the GRADE approach improved transparency regarding the certainty of evidence.

### 4.2. Study Limitations

Several limitations should be acknowledged. Only two RCTs evaluated ESR, limiting the strength and generalizability of conclusions for this outcome. Substantial heterogeneity (I^2^ = 93%) was observed in the CRP analysis, likely reflecting variations in intervention characteristics, participant profiles, and timing of outcome assessment across trials (4–29 weeks). Although inclusion was restricted to randomized controlled trials, some included studies exhibited methodological limitations that may have introduced performance or detection bias. All biomarker measurements were obtained immediately post-intervention, with no medium- or long-term follow-up; therefore, the durability of the observed CRP reductions remains uncertain. Finally, the relatively small number of included trials limits the robustness of subgroup and sensitivity analyses and precludes a reliable assessment of publication bias.

### 4.3. Implications for Future Research

Future RCTs should prioritize methodological rigor to enhance internal validity and reproducibility. Adequate random sequence generation and allocation concealment should be clearly reported, and assessor blinding should be implemented whenever feasible. The use of appropriate sham or comparator interventions tailored to physical therapy is recommended to minimize performance bias. Intention-to-treat analysis and transparent management of attrition are essential. Standardization of intervention protocols and dosing parameters, along with scientifically justified sample size calculations and inclusion of long-term follow-up assessments, will be critical to reducing heterogeneity and clarifying the clinical relevance of biomarker changes.

## 5. Conclusions

This systematic review and meta-analysis suggests that physical therapy is associated with reduced CRP levels in individuals with knee OA, whereas no clear effect was observed for ESR. However, substantial heterogeneity and the limited number of trials warrant cautious interpretation. As most included studies evaluated multimodal interventions, the independent effects of specific physical therapy modalities remain unclear. Future high-quality RCTs with standardized protocols and clearly defined dosing parameters are required to clarify the role of physical therapy in modulating inflammatory biomarkers in knee OA.

## Figures and Tables

**Table 2:** Outcomes and main findings of the included studies.

Author (Year)	Biomarkers	Other Outcomes	Findings (CRP/ESR)	Findings (Other Outcomes)
Kelmendi et al. (2024) [19]	CRP	Leptin, Adiponectin, Total Cholesterol, LDL	No changes at Week 29	No changes in all biomarkers
Lee et al. (2023) [20]	CRP, ESR	FTSST, TUG, strength, VAS	No change in CRP or ESR	EG > CG in FTSST; TUG ↓ and strength ↑ in EG; VAS ↓ in CG
Lin et al. (2023) [23]	CRP	WOMAC, VAS	Both improved; EG > CG	WOMAC and VAS improved; EG > CG
Samut et al. (2015) [21]	CRP	VAS, WOMAC, 30 s STS, 6 MWT	Both EGs improved > CG (not significant)	EGs improved vs. baseline; CG no change
Shabbir et al. (2022) [8]	CRP	NPRS, WOMAC	Both groups improved	EG > CG
Wei et al. (2024) [22]	CRP, ESR	VAS, LKSS	Both improved; EG > CG	EG had lower VAS and higher LKSS
Zhang et al. (2013) [24]	CRP	KJIS	CRP improved; EG > CG	KJIS improved; EG > CG

Abbreviations: 30 s STS, 30-Second Sit-to-Stand Test; 6 MWT, 6-Minute Walk Test; CG, comparison group; CRP, C-reactive protein; EG, experimental group; ESR, erythrocyte sedimentation rate; FTSST, Five-Times Sit-to-Stand Test; KJIS, Knee Joint Index Score; LDL, low-density lipoprotein; LKSS, Lysholm Knee Scoring Scale; NPRS, Numeric Pain Rating Scale; TUG, Timed Up and Go Test; VAS, Visual Analog Scale; WOMAC, Western Ontario and McMaster Universities Osteoarthritis Index; >, greater than; ↑, increase; ↓, decrease.

**Table 3:** Methodological quality assessment of the included randomized controlled trials using the PEDro scale.

Author, Year Criteria:	1	2	3	4	5	6	7	8	9	10	11	Total
Kelmendi et al. (2024) [19]	Y	Y	Y	Y	N	N	Y	Y	Y	Y	Y	8
Lee et al. (2023) [20]	Y	Y	Y	Y	N	N	Y	Y	Y	Y	Y	8
Lin et al. (2023) [23]	Y	Y	N	Y	N	N	N	Y	Y	Y	Y	6
Samut et al. (2015) [21]	N	Y	N	Y	N	N	N	Y	N	Y	Y	5
Shabbir et al. (2022) [8]	Y	Y	Y	Y	N	N	Y	Y	Y	Y	Y	8
Wei et al. (2024) [22]	Y	Y	Y	Y	Y	N	N	Y	N	Y	Y	7
Zhang et al. (2013) [24]	Y	Y	N	Y	N	N	N	Y	N	Y	Y	5

Y: yes, N: no. Criteria: 1 = eligibility criteria; 2 = random allocation; 3 = concealed allocation; 4 = baseline similarity; 5 = blinding of subjects; 6 = blinding of therapists; 7 = blinding of assessors; 8 = measure key outcomes > 85% of subjects; 9 = intention-to-treat analysis; 10 = between-group statistical comparisons; 11 = point estimates and variability.

**Table 4:** Summary of findings and GRADE certainty assessment for inflammatory biomarkers.

Certainty Assessment							Summary of Findings		
Participants (Studies) Follow-Up	Risk of Bias	Inconsistency	Indirectness	Imprecision	Publication Bias	Overall Certainty of Evidence	Study Event Rates (%)		Anticipated Absolute Effects
							with Control or Usual Care	with Physical Therapy	Risk Difference with Physical Therapy
**C-reactive protein (follow-up: range 4 weeks to 29 weeks)**									
523 (7 RCTs)	not serious	serious ^a^	not serious	not serious	none	⨁⨁⨁◯ Moderate ^a^	254	269	SMD 1.05 SD lower (1.80 lower to 0.31 lower)
**Erythrocyte Sedimentation Rate (follow-up: range 4 weeks to 8 weeks)**									
139 (2 RCTs)	not serious	serious	not serious	serious ^c^	none	⨁⨁◯◯ Low ^b,c^	70	69	SMD 0.2 SD lower (0.84 lower to 0.45 higher)

SMD: standardized mean difference. Explanations: ^a^ downgraded one level due to considerable statistical heterogeneity (I^2^ = 93%). Although subgroup and sensitivity analyses reduced heterogeneity, substantial variability remained across studies. ^b^ Downgraded one level due to moderate statistical heterogeneity (I^2^ = 63%) across the two included trials. ^c^ Downgraded one level due to wide confidence intervals crossing the line of no effect and a small total sample size (*n* = 139). ⊕⊕⊕⊕ High certainty ⊕⊕⊕◯ Moderate certainty ⊕⊕◯◯ Low certainty ⊕◯◯◯ Very low certainty.

## Data Availability

No new data were created or analyzed in this study. Data sharing is not applicable to this article. All data analyzed in this study are included in the published articles cited in the reference list.

## Supplementary Materials

The following supporting information can be downloaded at: https://www.mdpi.com/article/10.3390/jcm15135288/s1, PRISMA 2020 Checklist.

## Abbreviations

The following abbreviations are used in this manuscript:

30 s STS	30-Second Sit-to-Stand
6 MWT	6-Minute Walk Test
CI	Confidence Interval
CRP	C-reactive Protein
ESR	Erythrocyte Sedimentation Rate
FTSST	Five-Times Sit-to-Stand Test
GRADE	Grading of Recommendations Assessment, Development and Evaluation
GDT	Guideline Development Tool
I^2^	Inconsistency Statistic
IL-1β	Interleukin-1 Beta
LKSS	Lysholm Knee Scoring Scale
MeSH	Medical Subject Headings
NPRS	Numeric Pain Rating Scale
OA	Osteoarthritis
PEDro	Physiotherapy Evidence Database
PICOS	Population, Intervention, Comparator, Outcomes, and Study design
PRISMA	Preferred Reporting Items for Systematic Reviews and Meta-Analyses
PROSPERO	International Prospective Register of Systematic Reviews
RCT	Randomized Controlled Trial
RevMan	Review Manager
ROM	Range of Motion
SMD	Standardized Mean Difference
TNF-α	Tumor Necrosis Factor-Alpha
TUG	Timed Up and Go
VAS	Visual Analog Scale
WB-EMS	Whole-Body Electromyostimulation
WOMAC	Western Ontario and McMaster Universities Osteoarthritis Index
