# Supplementary material for: Effect of Physical Therapy on Inflammatory Biomarkers in Knee Osteoarthritis: A Systematic Review and Meta-Analysis of Randomized Controlled Trials
Source: J Clin Med. 2026 Jul 7;15(13):5288. doi: 10.3390/jcm15135288 (PMC13363646; doi:10.3390/jcm15135288)
Supplement: Supplementary file 1 [file jcm-15-05288-s001.zip › jcm-4394282-supplementary.pdf]

PRISMA 2020 Checklist

Section and Topic	Item #	Checklist item	Location where item is reported
**TITLE**			
Title	1	Identify the report as a systematic review.	Page 1
**ABSTRACT**			
Abstract	2	See the PRISMA 2020 for Abstracts checklist.	Page 1
**INTRODUCTION**			
Rationale	3	Describe the rationale for the review in the context of existing knowledge.	Last paragraph of the Introduction section (page 2)
Objectives	4	Provide an explicit statement of the objective(s) or question(s) the review addresses.	Last paragraph of the Introduction section (page 2)
**METHODS**			
Eligibility criteria	5	Specify the inclusion and exclusion criteria for the review and how studies were grouped for the syntheses.	Eligibility Criteria (Page 3)
Information sources	6	Specify all databases, registers, websites, organisations, reference lists and other sources searched or consulted to identify studies. Specify the date when each source was last searched or consulted.	Search Strategy (Page 2)
Search strategy	7	Present the full search strategies for all databases, registers and websites, including any filters and limits used.	Search Strategy (Page 2); Appendix (Page 14)
Selection process	8	Specify the methods used to decide whether a study met the inclusion criteria of the review, including how many reviewers screened each record and each report retrieved, whether they worked independently, and if applicable, details of automation tools used in the process.	Study Selection (Page 3)
Data collection process	9	Specify the methods used to collect data from reports, including how many reviewers collected data from each report, whether they worked independently, any processes for obtaining or confirming data from study investigators, and if applicable, details of automation tools used in the process.	Data Extraction; and Data Synthesis and Analysis (Page 3)
Data items	10a	List and define all outcomes for which data were sought. Specify whether all results that were compatible with each outcome domain in each study were sought (e.g. for all measures, time points, analyses), and if not, the methods used to decide which results to collect.	Data Extraction and Synthesis (Page 3); Description of included studies (Page 5)
	10b	List and define all other variables for which data were sought (e.g. participant and intervention characteristics, funding sources). Describe any assumptions made about any missing or unclear information.	Data extracted included study and participant characteristics, interventions, and outcomes (p. 3; Table 1 pp. 6–7). No assumptions made for missing data.
Study risk of bias assessment	11	Specify the methods used to assess risk of bias in the included studies, including details of the tool(s) used, how many reviewers assessed each study and whether they worked independently, and if applicable, details of automation tools used in the process.	Assessed independently by two reviewers using PEDro; consensus with third reviewer. No automation. (p. 3; Table3 p. 8)
Effect measures	12	Specify for each outcome the effect measure(s) (e.g. risk ratio, mean difference) used in the synthesis or presentation of results.	Data Synthesis & Analysis (Page 3)
Synthesis methods	13a	Describe the processes used to decide which studies were eligible for each synthesis (e.g. tabulating the study intervention characteristics and comparing against the planned groups for each synthesis (item #5)).	Eligibility Criteria (Page 3)
	13b	Describe any methods required to prepare the data for presentation or synthesis, such as handling of missing summary statistics, or data conversions.	Data were checked for consistency; all RCTs reported

PRISMA 2020 Checklist

Section and Topic	Item #	Checklist item	Location where item is reported
			outcomes in comparable units, and no conversions or imputations were applied prior to synthesis (page 3).
	13c	Describe any methods used to tabulate or visually display results of individual studies and syntheses.	Study characteristics (Table 1and 2, pp. 6–7) and risk-of-bias assessment (Table 3, p. 8) were tabulated; forest plots (Figures 2 and 3, p. 9) in RevMan 5 displayed individual and pooled effect estimates with 95% CIs and heterogeneity.
	13d	Describe any methods used to synthesize results and provide a rationale for the choice(s). If meta-analysis was performed, describe the model(s), method(s) to identify the presence and extent of statistical heterogeneity, and software package(s) used.	Meta-analysis performed using a random-effects model (RevMan 5). Heterogeneity assessed with I^2 statistic. SMDs and 95% CIs calculated. Random-effects model chosen due to expected clinical/methodological variability (Page 3).
	13e	Describe any methods used to explore possible causes of heterogeneity among study results (e.g. subgroup analysis, meta-regression).	Subgroup analysis by number of physical-therapy sessions per day used to explore heterogeneity (Page 3).
	13f	Describe any sensitivity analyses conducted to assess robustness of the synthesized results.	In results section. Leave-one-out sensitivity analysis showed stable pooled results
Reporting bias assessment	14	Describe any methods used to assess risk of bias due to missing results in a synthesis (arising from reporting biases).	Not assessed due to limited number of included studies (< 10).
Certainty assessment	15	Describe any methods used to assess certainty (or confidence) in the body of evidence for an outcome.	Certainty of evidence GRADE in result section.
**RESULTS**			
Study selection	16a	Describe the results of the search and selection process, from the number of records identified in the search to the number of studies included in the review, ideally using a flow diagram.	presented in PRISMA flow diagram (Figure 1, p. 4), showing 7 included RCTs.
	16b	Cite studies that might appear to meet the inclusion criteria, but which were excluded, and explain why they were excluded.	Excluded full-texts (non-RCTs, irrelevant treatments or outcomes, or lacking mean and SD) detailed in the PRISMA flow diagram (Figure 1, p. 4).
Study characteristics	17	Cite each included study and present its characteristics.	Characteristics of included RCTs (n = 7; refs 9, 15–20) are summarized in Table 1 (pp. 6–7)

PRISMA 2020 Checklist

Section and Topic	Item #	Checklist item	Location where item is reported
			and described in Section 3.2 (p. 5).
Risk of bias in studies	18	Present assessments of risk of bias for each included study.	Risk-of-bias results shown in Table 3 and Section 3.3 (p. 8).
Results of individual studies	19	For all outcomes, present, for each study: (a) summary statistics for each group (where appropriate) and (b) an effect estimate and its precision (e.g. confidence/credible interval), ideally using structured tables or plots.	Results for CRP, ESR, and other outcomes shown in Table 2 (pp. 6–7), Section 3.4 (pp. 8–9), and forest plots (Figures 2–3, p. 9).
Results of syntheses	20a	For each synthesis, briefly summarise the characteristics and risk of bias among contributing studies.	For CRP and ESR syntheses, study characteristics summarized in Table 2 (pp. 6–7) and risk-of-bias results in Table 3 (Section 3.3, p. 8)
	20b	Present results of all statistical syntheses conducted. If meta-analysis was done, present for each the summary estimate and its precision (e.g. confidence/credible interval) and measures of statistical heterogeneity. If comparing groups, describe the direction of the effect.	Meta-analysis results presented in Section 3.4 (pp. 8–9) and Figures 2–3 (p. 9).
	20c	Present results of all investigations of possible causes of heterogeneity among study results.	Heterogeneity explored by session frequency (Section 3.4, pp. 8–9) and discussed in Section 4 (p. 10).
	20d	Present results of all sensitivity analyses conducted to assess the robustness of the synthesized results.	Sensitivity analysis showed stable results with no substantial change in pooled estimates.
Reporting biases	21	Present assessments of risk of bias due to missing results (arising from reporting biases) for each synthesis assessed.	Reporting bias not assessed due to limited number of studies (< 10)
Certainty of evidence	22	Present assessments of certainty (or confidence) in the body of evidence for each outcome assessed.	The certainty of evidence ranged from low to moderate according to GRADE assessment.
**DISCUSSION**			
Discussion	23a	Provide a general interpretation of the results in the context of other evidence.	Discussion section (p. 9-10).
	23b	Discuss any limitations of the evidence included in the review.	Last paragraph of the Discussion (p. 10).
	23c	Discuss any limitations of the review processes used.	Last paragraph of the Discussion (p. 10).
	23d	Discuss implications of the results for practice, policy, and future research.	Discussion section (p. 9-10).
**OTHER INFORMATION**			
Registration and protocol	24a	Provide registration information for the review, including register name and registration number, or state that the review was not registered.	Registered in PROSPERO (CRD42024590843, 15 Nov 2024); see Section 2.1 (p. 2).
	24b	Indicate where the review protocol can be accessed, or state that a protocol was not prepared.	Protocol available in PROSPERO (CRD42024590843);

PRISMA 2020 Checklist

Section and Topic	Item #	Checklist item	Location where item is reported
			see Section 2.1 (p. 2).
	24c	Describe and explain any amendments to information provided at registration or in the protocol.	No amendments made to the registered PROSPERO protocol (CRD42024590843).
Support	25	Describe sources of financial or non-financial support for the review, and the role of the funders or sponsors in the review.	Conducted without external funding (Funding statement, p. 11).
Competing interests	26	Declare any competing interests of review authors.	Authors declare no conflicts of interest (Conflicts of Interest, p. 11).
Availability of data, code and other materials	27	Report which of the following are publicly available and where they can be found: template data collection forms; data extracted from included studies; data used for all analyses; analytic code; any other materials used in the review.	All data available within the article (Data Availability Statement, p. 11).

From: Page MJ, McKenzie JE, Bossuyt PM, Boutron I, Hoffmann TC, Mulrow CD, et al. The PRISMA 2020 statement: an updated guideline for reporting systematic reviews. BMJ 2021;372:n71. doi: 10.1136/bmj.n71.
This work is licensed under CC BY 4.0. To view a copy of this license, visit https://creativecommons.org/licenses/by/4.0/
